# Nailfold capillary patterns in ballgame and endurance athletes

**DOI:** 10.3389/fphys.2025.1568972

**Published:** 2025-04-16

**Authors:** Takeshi Otsuki, Kazuya Suwabe, Toru Yoshikawa, Kiwamu Kotani, Asako Zempo-Miyaki

**Affiliations:** Faculty of Sport and Health Sciences, Ryutsu Keizai University, Ryugasaki, Japan

**Keywords:** basketball, light microscopy, long-distance runner, soccer, triathlete

## Abstract

**Purpose:**

Nailfold capillary patterns can be observed noninvasively using light microscopy. Nailfold capillaries are straight, U-shaped, and densely looped in healthy individuals and may be altered by disease or lifestyle factors, such as diet. However, the effects of daily physical activity and exercise training on nailfold capillary patterns remain unclear. This study aimed to examine the effects of exercise training on nailfold capillary patterns by investigating these patterns in endurance athletes, ballgame athletes, and sedentary healthy men.

**Methods:**

Five healthy men participated in nailfold capillary pattern measurements on three consecutive days to test the reproducibility and bilateral differences in the nailfold capillary loop density, length, and width measured using light microscopy and a commercial analysis system. The nailfold capillaries of 10 endurance athletes (endurance group; eight long-distance runners and two triathletes), 10 ballgame athletes (ballgame group; seven soccer players and three basketball players), and nine sedentary healthy men (sedentary group) were then examined using light microscopy.

**Results:**

The day-to-day coefficient of variation for the nailfold capillary loop density, length, and width were 4.9% ± 1.6%, 7.5% ± 1.3%, and 4.2% ± 1.5%, respectively. No significant differences in these measurements were observed between the dominant and non-dominant hands. Nailfold capillary density was greater in the ballgame group than in the endurance and sedentary groups. Capillary loop length was shorter in the ballgame group than in the endurance and sedentary groups. No significant differences in capillary loop density and length were observed between the endurance and sedentary groups. No significant intergroup differences were observed in capillary loop width.

**Conclusion:**

These results suggest that the nailfold capillary patterns of male ballgame athletes differ from those of endurance athletes and sedentary healthy men. Ballgame training may affect nailfold capillary patterns in sedentary healthy men.

## 1 Introduction

Exercise training affects capillaries in the skeletal muscle. Denis et al. (1986) showed that capillary density increases with endurance training. Tesch et al. (1984) reported that the capillary density in the vastus lateralis muscle was lower in weight/power lifters than in endurance runners and nonathletes. In an animal study, Adolfsson et al. (1981) demonstrated that swimming increased capillary density in skeletal muscle. These findings suggest that capillary development in skeletal muscle plays a role in improving endurance capacity in response to endurance training. The adaptation of skeletal muscle capillaries to exercise training is an interesting research topic that may have clinical relevance, such as identifying athletes with exceptional endurance potential and evaluating the effects of endurance training. However, previous studies used invasive measurement methods such as muscle biopsy (Denis et al., 1986; Tesch et al., 1984) which limited their use for research purposes.

Nailfold capillary patterns can be observed noninvasively using light microscopy (Grover et al., 2022; Smith et al., 2023) and may serve as a surrogate for assessing skeletal muscle capillaries. First, the nailfold is part of the skin and, like skeletal muscle, contains continuous capillaries. Second, blood-flow-induced shear stress plays a crucial role in vascular adaptation (Kruger-Genge et al., 2019), and blood flow increases during exercise in both the skin and skeletal muscle (Armstrong et al., 1987). Nailfold capillaries are straight, U-shaped, and densely looped in healthy individuals (Ingegnoli et al., 2013) but may be altered by diseases, such as diabetes and hypertension (Suma et al., 2023), and other factors such as *Pantoea agglomerans* intake (Nakata et al., 2018). Notably, in diabetes, several previous studies have reported that abnormal nailfold capillary patterns are associated with diabetic retinopathy (Bakirci et al., 2019; Okabe et al., 2024; Shikama et al., 2021). If the effects of exercise training on nailfold capillary patterns are elucidated, they may become a useful index for the condition of athletes and the promotion of health in middle-aged and older individuals. Nakajima et al. (2022) recently reported that the nailfold capillary loop width was negatively correlated with the frequency of vigorous exercise in men aged ≥40 years. However, in that study (Nakajima et al., 2022), participants were only asked to report how often they vigorously exercised on a scale from 1 (low) to 5 (high). Further research is required to explain the relationship between exercise habits and nailfold capillary patterns. As a first step to elucidating the effects of athletic training on nailfold capillary patterns, this study investigated nailfold capillary patterns in endurance athletes, ballgame athletes, and sedentary healthy men. Endurance and ballgame athletes were chosen because previous studies have reported higher skeletal muscle capillary density in these populations (Randers et al., 2014). This study selected nailfold capillary loop density, length, and width as indices. Nailfold capillary loop density is a fundamental quantitative parameter in capillaroscopy studies, and decreased density, along with increased loop length or width, has been suggested as an indicator of diseases associated with capillary pathologies (Etehad Tavakol et al., 2015). These three indices can be measured using commercially available devices and software, making them practical for field applications. Additionally, we evaluated their reproducibility and bilateral consistency to ensure reliability for future research and field applications.

## 2 Materials and methods

### 2.1 Participants

Five healthy men (age, 27 ± 5 years; height, 171 ± 3 cm; and body weight, 61 ± 2 kg) participated in nailfold capillary pattern measurements on three consecutive days to test the measurement reproducibility of the capillary loop density, length, and width using light microscopy and a commercial analysis system. Since the fourth finger is less susceptible to physical stress than the second and third fingers, it was selected for observation (Nakajima et al., 2022; Nakata et al., 2018). Furthermore, the fourth fingers of their right and left hands were compared to assess the differences between the dominant and non-dominant hands.

Thereafter, 10 male endurance athletes (endurance group; eight long-distance runners and two triathletes), 10 male ballgame athletes (ballgame group; seven soccer players and three basketball players), and nine sedentary healthy men (sedentary group) participated in the capillary measurements. The athletes’ training habits are presented in Table 1. The sedentary healthy men had a sedentary lifestyle (no regular physical activity except for physical education classes) for at least 1 year. All participants were nonsmokers, were not taking any medication, and had no chronic diseases. Participants abstained from alcohol consumption on the day before the test and avoided caffeine consumption on the day of the study. In addition, they did not exercise, eat, or drink anything other than water for 2 h before the measurements.

**TABLE 1:** Physical characteristics, hemodynamics, and training habits of the participants.

	Sedentary (*n* = 9)			Endurance (*n* = 10)			Ballgame (*n* = 10)			ANOVA (*P*-value)
Age, years	21.1	±	0.6	20.1	±	0.3	20.1	±	0.4	0.20
Height, cm	173	±	2	173	±	2	175	±	2	0.84
Body weight, kg	62	±	2	59	±	2	67	±	2^†^	0.01
Body mass index, kg/m^2^	20.7	±	0.5	19.6	±	0.5	22.0	±	0.5^†^	<0.01
Body fat, %	13.4	±	1.2	8.9	±	0.5^*^	13.4	±	0.7^†^	<0.01
SBP, mmHg	127	±	2	117	±	3^*^	118	±	2^*^	0.01
DBP, mmHg	81	±	2	71	±	1^*^	77	±	2^†^	<0.01
Pulse rate, bpm	71	±	3	57	±	2^*^	65	±	3^†^	<0.01
Training time, h/day		-		3.3	±	0.6	2.3	±	0.3	0.14
RPE		-		14.8	±	0.6	14.4	±	0.6	0.65
Frequency, days/week		-		5.7	±	0.3	4.9	±	0.5	0.18
Career, years		-		9.5	±	1.3	11.1	±	1.0	0.35
Capillary density, loops/mm	7.2	±	0.5	7.2	±	0.5	9.1	±	0.6^*,†^	0.03
Capillary loop length, μm	249	±	22	244	±	21	174	±	21^*,†^	0.02
Capillary loop width, μm	43	±	2	43	±	3	39	±	1	0.30

**FIGURE 1:**
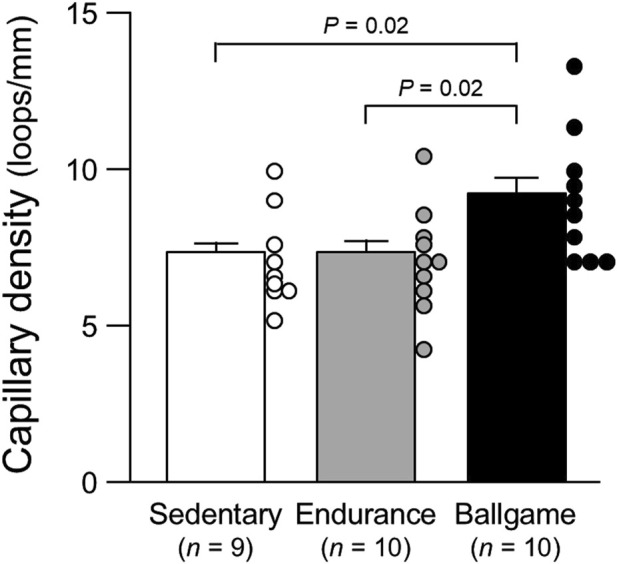
Nailfold capillary density in sedentary men, endurance athletes, and ballgame athletes Bars represent means ± standard errors, while circles indicate individual values.

**FIGURE 2:**
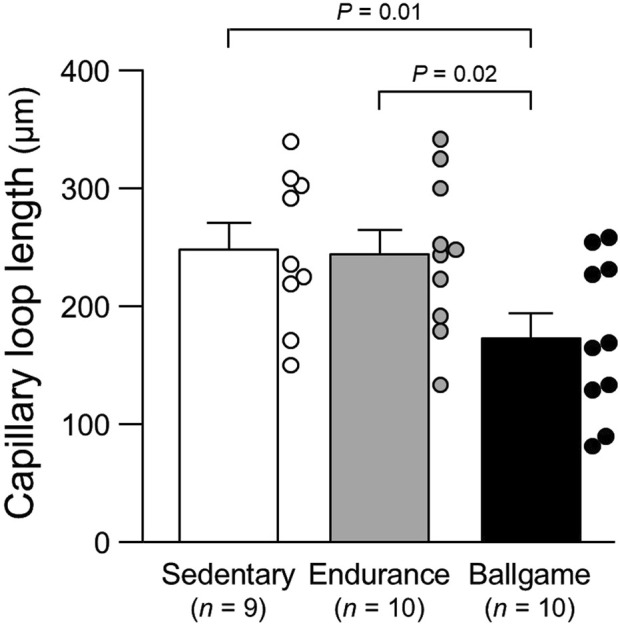
Nailfold capillary loop length in sedentary men, endurance athletes, and ballgame athletes. Bars represent means ± standard errors, while circles indicate individual values.

**FIGURE 3:**
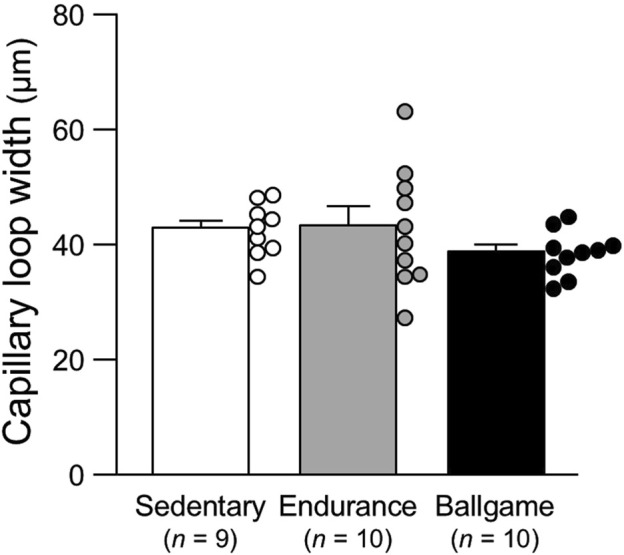
Nailfold capillary loop width in sedentary men, endurance athletes, and ballgame athletes. Bars represent means ± standard errors, while circles indicate individual values.

ANOVA, analysis of variance; SBP, systolic blood pressure; DBP, diastolic blood pressure; and RPE, rating of perceived exertion of usual training sessions (Borg’s 6–20 scale).*, *P* < 0.05 vs. sedentary healthy men and †, *P* < 0.05 vs. endurance athletes. The capillary data are presented also in Figures 1–3.

A power calculation was performed for one-way analysis of variance using G*Power 3 (Faul et al., 2007). The sample size in this study was sufficient to detect differences among the three groups with 90% power and an α of 5%, assuming an effect size of 0.8.

This study adhered to the tenets of the Declaration of Helsinki and was approved by the Ethics Committee of Ryutsu Keizai University (approval no. 34). All the participants provided written informed consent before participating in the study.

### 2.2 Nailfold capillaroscopy

In accordance with previous studies (Nakajima et al., 2022; Nakata et al., 2018), the fourth finger of the left hand was examined using commercially available light microscopy, after the application of mineral oil to reduce light reflection (Kekkan-Bijin; AT Co. Ltd., Osaka, Japan). Capillary images of the midpoint of the nailfold were captured automatically after focusing (CAS, AT Co. Ltd.). Additionally, adjacent left and right images were also captured. The number, length, and width of capillary loops within a specified field (500 × 700 μm and 480 × 640 pixels) were measured automatically using a commercial analysis system (CAS rating, AT Co. Ltd.) (Nakata et al., 2018). Capillary density is defined as the number of capillaries per 1 mm length of the distal row of each finger (Etehad Tavakol et al., 2015). The number of capillary loops within a 700 μm length was counted and then converted to the corresponding number per 1 mm length. Capillary loop length and width are defined as the distance from the apex of the loop to the point where the capillary was no longer visible and the length of its widest part, respectively (Etehad Tavakol et al., 2015). The length and width of each capillary loop within the images were measured, and the mean values from the three images were calculated. On days 2 and 3, to assess reproducibility, images from the previous day were used as references to ensure that capillary images were captured from the same field of view.

### 2.3 Blood pressure, pulse rate, and body fat

The brachial arterial blood pressure and pulse rate were measured in duplicate in the sitting position (UA-782; Nissei, Shibukawa, Japan). Body weight and fat content were measured using a body impedance-based body composition analyzer (InBody 430; InBody, Seoul, South Korea).

### 2.4 Statistical analysis

Values are expressed as the mean ± standard error. Unpaired *t*-tests were used for comparisons between the dominant and non-dominant hands. Between-group differences were tested using analysis of variance. When a significant *F*-value was observed, Fisher’s *post hoc* test was performed. *P* < 0.05 was considered statistically significant.

## 3 Results

Nailfold capillary loop density on days 1, 2, and three were 7.9 ± 0.4, 7.8 ± 0.5, and 7.7 ± 0.8 loops/mm, respectively. Similarly, the capillary loop length was 201 ± 32, 219 ± 33, and 222 ± 36 μm on days 1, 2, and 3, respectively. Likewise, the capillary loop width measured 44 ± 2, 43 ± 2, and 44 ± 3 μm on the corresponding days. The day-to-day coefficient of variation for the nailfold capillary loop density, length, and width were 4.9% ± 1.6%, 7.5% ± 1.3%, and 4.2% ± 1.5%, respectively. No significant differences in the capillary loop density (8.0 ± 0.8 vs. 7.9 ± 0.4 loops/mm, *P* = 0.97), length (187 ± 19 vs. 201 ± 32 μm, *P* = 0.71), and width (44 ± 2 vs. 44 ± 2 μm, *P* = 0.90) were observed between the dominant and non-dominant hands.

None of the participants habitually consumed alcohol. Furthermore, no significant differences in habitual sleep duration were observed among the sedentary, endurance, and ballgame groups (367 ± 18 vs. 405 ± 14 vs. 420 ± 13 min/day, *P* = 0.06). Body weights and body mass index (BMI) were higher in the ballgame athletes than in the endurance athletes (Table 1). No participants had a BMI exceeding 25 kg/m^2^. Body fat was lower in the endurance group than in the sedentary and ballgame groups. Systolic blood pressure was lower in the endurance and ballgame athletes than in the sedentary healthy men. The diastolic blood pressure and pulse rate were lower in the endurance athletes than in the sedentary healthy men and ballgame athletes.

Nailfold capillary loop density was greater in the ballgame group than in the endurance and sedentary groups (Table 1; Figure 1). Capillary loop length was shorter in ballgame athletes than in endurance athletes and their sedentary peers (Figure 2). No significant differences in capillary loop width were observed between the groups (Figure 3). The same results were obtained when the triathletes and basketball players were excluded from the analyses; the soccer players had a higher density and shorter length of capillary loops than the long-distance runners and sedentary participants (*P* ≤ 0.01).

## 4 Discussion

This study investigated nailfold capillary patterns in endurance athletes, ballgame athletes, and sedentary healthy men. First, the reproducibility and bilateral differences in the nailfold capillary loop density, length, and width measured using light microscopy and a commercial analysis system were tested. The day-to-day coefficients of variation of these indices ranged from 4.2% to 7.5%. No significant differences in these measurements were observed between the dominant and non-dominant hands. Next, we examined the nailfold capillary patterns in male long-distance runners, triathletes, soccer players, basketball players, and sedentary healthy men. We found that the nailfold capillary density was higher and that the loop length was shorter in ballgame athletes than in endurance athletes and their sedentary peers. These results suggest that the capillary patterns of male ballgame athletes differ from those of endurance athletes and sedentary healthy men. Moreover, exercise training for ballgames may affect nailfold capillary patterns in healthy young men.

The nailfold is part of the skin, and both the skin and skeletal muscle contain continuous capillaries. However, their primary functions differ: skin capillaries primarily regulate thermoregulation, whereas skeletal muscle capillaries facilitate the delivery of essential substances (Heistad and Abboud, 1974). As a result, blood flow in skeletal muscle increases proportionally with exercise intensity, whereas in the skin, it decreases during high-intensity exercise compared to moderate-intensity exercise (Armstrong et al., 1987). Blood flow is a key regulator of vascular endothelial growth factor (VEGF) receptor expression (Zhou et al., 2014), and VEGF is considered a crucial factor in capillary pattern changes (Emrani et al., 2017). This suggests that nailfold capillary patterns may not directly reflect the hemodynamics of skeletal muscles. However, skin blood flow remains higher during high-intensity exercise than at rest. Additionally, exercise affects blood flow and blood pressure not only in the active limbs but also in the inactive limbs. Tanaka et al. (2006) showed that blood flow increased in the brachial artery during leg cycling exercises and in the femoral artery during arm cycling exercises. Blood pressure also increases in the inactive arm during arm curl exercise of the contralateral arm (Otsuki et al., 2016) and leg press exercise (Otsuki and Kotato, 2019). Therefore, systemic circulatory responses associated with intensity of training for soccer and basketball may cause changes in nailfold capillary patterns.

Blood-flow-induced shear stress is an important factor in the initiation of vascular adaptation (Kruger-Genge et al., 2019). Maximal oxygen uptake is greater in endurance athletes than in ballgame athletes (Degens et al., 2019), and blood flow and shear stress during exercise are also thought to be greater in endurance athletes. Before conducting this study, we hypothesized that nailfold capillary adaptations would be greater in endurance athletes than in ballgame athletes. However, the only group that had a capillary pattern different from that of sedentary healthy men were the ballgame athletes. Ballgames, such as soccer and basketball, are considered to be intermittent exercise, as opposed to long-distance running and triathlon, which are considered to be continuous exercise. This means that ballgame athletes repeat high-, moderate-, and low-intensity exercises and rest during the games and training. Blood flow and oxygen consumption change proportionally to exercise intensity (Otsuki et al., 2006). Although this is only speculation, short and numerous capillaries may be suitable for nailfold blood circulation during intermittent exercise in which the rapid regulation of blood flow is repeated.

Ballgame athletes perform both endurance and strength training. Differences exist between the vascular adaptations of endurance and strength training. The skeletal muscle capillary density increases during endurance training (Denis et al., 1986) and decreases during strength training (Tesch et al., 1984). Arterial stiffness is lower in endurance athletes and higher in high-strength athletes (Otsuki et al., 2007). There may also be differences in the nailfold capillary adaptations that occur with endurance and strength training. Further studies on nailfold capillary patterns in strength-trained athletes should be performed to determine whether the unique nailfold capillary patterns of ballgame athletes are a result of strength training or other factors such as the intermittent nature of ball playing.

Previous studies have identified various factors that can influence nailfold capillary morphology. Individuals aged 40 or 41 years and older have been reported to exhibit higher capillary density than younger individuals, which may represent an adaptation to declining capillary function (Hoerth et al., 2012; Nakajima et al., 2022). However, healthy individuals generally have greater capillary density than those suspected of having capillary pathologies within the same age group (Hoerth et al., 2012). Additionally, a conflicting result has been reported, indicating no significant difference in capillary density between individuals over 41 years and those under 40 years (Gorasiya et al., 2022). A higher BMI (>25 kg/m^2^) was also linked to an increased prevalence of abnormal capillaries (Gorasiya et al., 2022). Smokers tend to exhibit increased capillary width (Nakajima et al., 2022; Yuksel et al., 2019). Additionally, increased capillary width was correlated with shorter time taken to fall asleep, while shorter capillary length was associated with higher alcohol consumption (Nakajima et al., 2022). However, in this study, all participants were young, non-smokers with a BMI of less than 25 kg/m^2^ and no habitual alcohol consumption. Additionally, no significant differences were observed among the groups in terms of age, BMI, or habitual sleep duration. Therefore, the observed differences in capillary morphology among the groups in this study are likely attributable to habitual exercise patterns.

This study has certain limitations. First, we could not investigate the mechanisms responsible for intergroup differences in nailfold capillary patterns. Second, strength athletes were not included in this study. Third, this was a cross-sectional study with a small cohort. Fourth, although various indices of capillary patterns exist, such as capillary diameter, internal diameter of capillary loop, and inter-capillary distance (Etehad Tavakol et al., 2015), we measured only capillary density, length, and width. The conclusions of this study should be verified through larger, longitudinal studies. Despite these limitations, we believe that this study is an important first step toward understanding the effects of athletic exercise training on nailfold capillary patterns.

In conclusion, the results of this study suggest that the nailfold capillary patterns of male ballgame athletes differ from those of male endurance athletes and sedentary healthy men. Furthermore, they demonstrate that ballgame training may affect nailfold capillary patterns in young men. Further research should be conducted to determine whether the unique nailfold capillary patterns of ballgame athletes are a result of strength training or other possible factors such as the intermittent nature of ball playing.

## Data Availability

The original contributions presented in the study are included in the article/supplementary material, further inquiries can be directed to the corresponding author.
